# RNA sequencing of *Brassica napus* reveals cellular redox control of *Sclerotinia* infection

**DOI:** 10.1093/jxb/erx338

**Published:** 2017-09-27

**Authors:** Ian J Girard, Chaobo Tong, Michael G Becker, Xingyu Mao, Junyan Huang, Teresa de Kievit, W G Dilantha Fernando, Shengyi Liu, Mark F Belmonte

**Affiliations:** 1 Department of Biological Sciences, University of Manitoba, Winnipeg, MB R3T 2N2, Canada; 2 Oil Crops Research Institute, Chinese Academy of Agricultural Sciences, The Key Laboratory of Biology and Genetic Improvement of Oil Crops, The Ministry of Agriculture, Wuhan 430062, Hubei, China; 3 Department of Microbiology, University of Manitoba, Winnipeg, MB R3T 2N2, Canada; 4 Department of Plant Science, University of Manitoba, Winnipeg, MB R3T 2N2, Canada

**Keywords:** *Brassica napus*, disease, oilseed rape, plant-pathogen interactions, RNA seq, redox, *Sclerotinia sclerotiorum*, transcription factor network

## Abstract

*Brassica napus* is one of the world’s most valuable oilseeds and is under constant pressure by the necrotrophic fungal pathogen, *Sclerotinia sclerotiorum*, the causal agent of white stem rot. Despite our growing understanding of host pathogen interactions at the molecular level, we have yet to fully understand the biological processes and underlying gene regulatory networks responsible for determining disease outcomes. Using global RNA sequencing, we profiled gene activity at the first point of infection on the leaf surface 24 hours after pathogen exposure in susceptible (*B. napus* cv. Westar) and tolerant (*B. napus* cv. Zhongyou 821) plants. We identified a family of ethylene response factors that may contribute to host tolerance to *S. sclerotiorum* by activating genes associated with fungal recognition, subcellular organization, and redox homeostasis. Physiological investigation of redox homeostasis was further studied by quantifying cellular levels of the glutathione and ascorbate redox pathway and the cycling enzymes associated with host tolerance to *S. sclerotiorum*. Functional characterization of an Arabidopsis redox mutant challenged with the fungus provides compelling evidence into the role of the ascorbate-glutathione redox hub in the maintenance and enhancement of plant tolerance against fungal pathogens.

## Teaser

Protection against *Sclerotinia sclerotiorum* in *Brassica napus* is mediated via dynamic transcription factor networks and cellular redox homeostasis directly at the site of infection.

## Introduction

*Brassica napus* (oilseed rape) is the second most valuable oilseed crop in the world and is vulnerable to white stem rot caused by the necrotrophic ascomycete *Sclerotinia sclerotiorum*, one of the most devastating fungal crop pathogens (Bolton, 2006; Hegedus and Rimmer, 2005). Currently, control of *S. sclerotiorum* relies heavily on the application of broad-spectrum fungicides (Bradley *et al.*, 2006) as few resistant cultivars have been developed and microbial biocontrol methods, although promising, have yet to be implemented (Fernando *et al.*, 2007; Khot *et al.*, 2011). Developing new strategies to combat yield losses in *B. napus* requires a thorough understanding of the genes and gene regulatory networks underlying the plant defense response, especially in existing tolerant breeding cultivars (Garg *et al.*, 2010*a*).

*B. napus* is vulnerable to carpogenic attack from *S. sclerotiorum*; however, for the fungus to penetrate the host plant, ascospores require an external energy source (Garg *et al.*, 2010b; Hegedus and Rimmer, 2005; Mclean, 1958). Field-based evidence has shown that fungal colonization of petals may provide a nutrient source for producing the infection cushions required for penetrating mature leaves (Jamaux *et al.*, 1995; Huang *et al.* 2008). Unfortunately, all recent work directed at understanding the pathosystem uses either an artificial sugar-phosphate based ascospore assay (Garg *et al.*, 2013) or a myceliogenic infection using sclerotia germinated on fungal growth media (Wu *et al.*, 2016; Zhao *et al.*, 2009). As defense activation likely occurs at the petal inoculum-leaf interface, there is a need to understand global transcriptional responses directly at the infection site under conditions reflective of natural *Sclerotinia* disease transmission.

Plants have dynamic signalling networks to carefully balance growth and defense processes and maximize fitness (Huot *et al.*, 2014). Pathogen perception in the host is orchestrated in part through pathogen associated molecular pattern (PAMP) detection via pattern recognition receptors (PRRs). Following PRR activation, subsequent signal transduction to the nucleus transcriptionally reprograms the cell (Park and Ronald, 2012; Zipfel, 2014). PAMP detection elicits a signalling cascade through Ca^2+^ channel activation, nitric oxide signalling, reactive oxide species (ROS) bursts, and mitogen-activated protein kinase (MAPK) cascades (Boller and Felix, 2009; Meng and Zhang, 2013). Effective signal transduction leads to activation of transcription factors (TFs) that regulated bioprocesses responsible for plant defense (Schluttenhofer and Yuan, 2015; Tsuda and Somssich, 2015). Defense to necrotrophic fungi is partially controlled via jasmonic acid (JA)/ethylene (ET) hormone signalling (Denancé *et al.*, 2013). These hormones activate defense genes and stimulate production of antimicrobial compounds including glucosinolates and their derivatives (Wu *et al.*, 2014). Restriction of pathogen growth via PAMP signalling is referred to as PAMP-triggered immunity (PTI), and is generally synonymous with basal plant defense.

*Sclerotinia sclerotiorum* produces a suite of digestive enzymes to degrade its host, however its main pathogenicity factor is oxalic acid (OA, Cessna *et al.*, 2000). OA has a dual role of initially suppressing host cell ROS signalling, then subsequently eliciting ROS and plant cell death (Horbach *et al.*, 2011; Kim *et al.*, 2008; Williams *et al.*, 2011). The ascorbate-glutathione (ASC-GSH) pathway is at the core of the plant antioxidant system, which protects ROS signalling and reduces H_2_O_2_ toxicity (Foyer and Noctor, 2011; de Pinto *et al.*, 2012). Previous microarray-based experiments have implicated the antioxidant response in *B. napus* defense against *S. sclerotiorum* (Yang *et al.*, 2007); however, the physiological changes contributing to antioxidant-mediated defenses and the transcriptional regulation of these processes has yet to be studied.

Recent work profiling infected *B. napus* stem tissue has provided insight into the molecular processes that underlie defense to *S. sclerotiorum* (Wu *et al.*, 2016). For example, genes involved in glucosinolate biosynthesis and chitinase activity were highly upregulated early in the defense response in resistant *B. napus* (Wu *et al.*, 2016) and is consistent with findings in resistant *B. napus* leaves infected with the facultative necrotroph *Leptosphaeria maculans* (Becker *et al.* 2017a). In *B. napus* cv. Zhongyou821 (ZY821), a moderately tolerant cultivar, a putative resistance-associated quantitative trait locus has been identified as *INDOLE GLUCOSINOLATE METHYL TRANSFERASE 5* (Li *et al.*, 2006; Wu *et al.*, 2013). Activation of glucosinolate biosynthesis is transcriptionally controlled through gene regulatory networks at the leaf surface (Schweizer *et al.*, 2013; Sønderby *et al.*, 2010). Thus, thorough analyses of the transcriptional circuitry operative directly at the host pathogen interface at the leaf surface should identify regulatory elements that control disease progression.

In this study, we present a structural, molecular, and physiological investigation of the *B. napus* defense response to *S. sclerotiorum* in susceptible and tolerant hosts. The use of a petal inoculation method with *S. sclerotiorum* ascospores mimics disease transmission under real-world conditions. Global transcriptome profiling coupled with a comprehensive bioinformatic analysis revealed distinct biological processes not previously described in this system directly at the site of infection. Further, we used the latest information of *B. napus* TFs and promoter motifs to develop predictive gene regulatory networks responsible for disease tolerance in ZY821. This includes a putative ethylene response factor-controlled transcriptional circuit regulating redox state homeostasis genes that slows disease progression directly at and adjacent to the first site of infection in the leaf.

## Materials and methods

### *Brassica napus* growth conditions

The two *B. napus* cultivars used for all experiments are the susceptible *B. napus* L. cv. Westar (Westar) and *B. napus* L. cv. Zhongyou821 (ZY821). Westar seeds stored at 4°C were planted in Sunshine Mix No.1 soil and grown at 22°C with 50–70% humidity in a growth chamber with long day conditions (16 hours light, 8 hours dark 150–200 µE/m^2^/s). ZY821 plants were treated similarly, however 1 month after planting they were subjected to a four-week vernalization treatment (8 hours light, 16 hours dark, 40% humidity, 8–10°C and 100 µE/m^2^/s), before being transferred back to long day conditions. Both cultivars were inoculated at 30–50% bloom stage.

### *Sclerotinia sclerotiorum* inoculum preparation

*Sclerotinia sclerotiorum* field-collected ascospores were provided by Dr. K. Rashid, (Agriculture and AgriFood Canada, Morden, Manitoba), and stored at 4°C in desiccant in the dark. Inoculum was made by suspending ascospores at a concentration of 8 x10^4^ spores per mL in a 0.02% Tween80 (http://www.sigmaaldrich.com/) solution. Following, 30µL of the solution was pipetted onto senescing petals placed in a sterile empty petri plate and sealed with parafilm. The Tween80 solution without ascospores was applied to petals and used for a mock inoculation control. Petals were incubated for 72 hours prior to being used in the leaf inoculation experiments to allow ascospore germination.

### Leaf inoculation and tissue collection

At approximately 1PM, either mock-inoculated petals or infected petals and *S. Sclerotiorum* hyphae were placed onto healthy leaves and incubated in clear plastic bags to increase humidity and promote *S. sclerotiorum* infection. Lesion size was measured at 24, 48, 72, 96, and 120hpi (hours post inoculation). For RNA collection and redox assays, lesions and surrounding 1cm of healthy leaf tissue were excised and immediately frozen in liquid nitrogen. For each biological replicate, lesions were pooled from a minimum of 3 different plants and ground to a powder in liquid nitrogen.

### Light microscopy

Leaf tissues directly at the infection site were fixed in a solution of 2.5% glutaraldehyde and 1.6% paraformaldehyde in 1X PBS. Tissue processing, mounting, and imaging was carried out exactly as described in Chan and Belmonte (2013).

### Scanning electron microscopy

Fresh leaf tissues were mounted on aluminum stubs using double stick carbon tape. Images were taken of fresh leaves on a Hitachi TM-1000 Tabletop microscope and collected using the TM-1000 software.

### RNA Isolation and cDNA sequencing library synthesis

RNA was isolated using Invitrogen Plant RNA Purification Reagent, and subsequently treated with the Ambion Turbo DNA-free DNase kit per the manufacturer’s protocol (https://www.thermofisher.com). Quantity and purity were assessed spectrophotometrically and quality of RNA samples was verified using RNA Nano Chips on an Agilent 2100 Bioanalyzer (http://www.genomics.agilent.com/). Following, mRNA was isolated from the total RNA pool using the NEB Next® Poly(A) mRNA Magnetic Isolation Module (New England Biolabs, https://www.neb.ca/) per manufacturer’s instructions with the following modifications: all reaction volumes were halved and only 7.5µL of Oligo d(T)_25_ beads were used per sample. RNA sequencing libraries were prepared from isolated mRNA using the alternate HTR protocol (C2) described in Kumar *et al.* (2012) beginning at first strand cDNA synthesis with the following modifications: NEXTflex™ ChIP-Seq Barcodes (Bioo Scientific, http://www.biooscientific.com/) were used as adaptors for the adapter ligations and NEXTflex™ PCR Primer Mix was used for the library PCR enrichment. Library size was verified using a High Sensitivity DNA chip on an Agilent 2100 Bioanalyzer. Libraries were pooled and size selected using an E-Gel® SizeSelect™ 2% agarose gel (Life Technologies, www.thermofisher.com) isolating 250–500 base pair fragments. Fragments were sequenced using the Illumina HiSeq2000 platform at the Génome Québec Innovation Centre (Montreal, Canada, http://gqinnovationcenter.com/). Sequencing was performed in high output mode, producing 100bp single end reads.

### Bioinformatics pipeline

Raw Fastq files were cleaned using Trimmomatic (Bolger *et al.*, 2014), clipping adapter sequences, performing a 6 base head crop, clipping reads when the average base quality score fell below 30 in 4 base sliding window, and dropping remaining reads shorter than 50nt. The splice junction mapping software TopHat (v2.0.13, Trapnell *et al.*, 2012, http://ccb.jhu.edu/software/tophat/index.shtml) was used to align and map the trimmed reads to *B. napus* genome v5.0 (Chalhoub *et al.*, 2014, http://www.genoscope.cns.fr/brassicanapus/) and the *S. sclerotiorum* genome (Amselem *et al.*, 2011, https://www.ncbi.nlm.nih.gov/nuccore/NZ_AAGT00000000.1). Transcripts from each sample were assembled with Cufflinks (v2.2.1, https://github.com/cole-trapnell-lab/cufflinks) using the transcript annotation files to guide assembly. Individual samples’ transcript assemblies were merged with the reference. For a conservative and robust prediction of novel transcripts, the merged assembly was used to predict open reading frames with Transdecoder (v2.0.1, https://transdecoder.github.io/), and predicted genes were only kept if they were located within intergenic regions and the largest translated ORF had a blast hit using BLASTp (v2.2.30, http://blast.ncbi.nlm.nih.gov/) to Arabidopsis TAIR10 (http://arabidopsis.org), with an E-value cut off<1 × 10^-10^. Quantification of mapped reads was done with the Tuxedo package (v2.2.1, http://cole-trapnell-lab.github.io/cufflinks). This included differential expression analysis, and normalization of transcript abundances across samples in FPKM. Fuzzy k-means clustering analysis was performed per Belmonte *et al.* (2013). Following, selected gene sets were used as input into SeqEnrich (Becker *et al.*, 2017b), a program adapted for *B. napus* from ChipEnrich (Belmonte *et al.*, 2013). SeqEnrich contains the most current information on *B. napus* TFs, promoter motifs, and gene ontology (GO) available, and uses these data to produce predictive regulatory networks. Networks produced with SeqEnrich were visualized in Cytoscape (http://www.cytoscape.org/).

### Quantitative RT-PCR analysis

One microgram of total RNA from each biological replicate was used to construct cDNA libraries for quantitative real-time PCR (qPCR) using the Maxima First Strand Synthesis Kit (https://www.thermofisher.com/). qPCR was carried out using SsoFast Evagreen Supermix (http://www.bio-rad.com/), following manufacturer’s instructions, but proportionally adjusting to a 10µL reaction volume. Reaction steps were performed as follows: 95°C for 30 seconds, followed by 45 cycles of 95°C for 2 seconds and 60°C for 5 seconds. The ΔΔCt method, using the ubiquitin family protein *BnaC08g11930D* as a housekeeping gene in *B. napus*, was used to calculate fold changes between mock and *S. sclerotiorum* inoculated treatments in both cultivars. For Arabidopsis experiments, *EF1ALFA* (*AT5G60390*) was used as a housekeeping gene (Nicot *et al.*, 2005). Statistical significance was calculated using the Students t-test (*P*<0.05) and all primer sequences used are presented in Table S9.

### Enzyme assays

*S. sclerotiorum* inoculation and tissue collection was carried out as described above. Quantification of reduced and oxidized forms of ascorbate and glutathione were carried out per Zhang and Kirkham (1996). Enzymatic activity of ascorbate peroxidase (APX), monodehydroascorbate reductase (MDAR), dehydroascorbate reductase (DHAR), and glutathione reductase (GR) were carried out exactly as described in Belmonte and Stasolla (2009).

### Arabidopsis pathogenicity experiments

*Arabidopsis thaliana* and T-DNA insertion lines were obtained from The Arabidopsis Biological Resource Center (Ohio State University, https://abrc.osu.edu/). Seeds were surface sterilized using 75% ethanol and grown on Murashige and Skrooge medium (Phytotechnology Labratories) with 1% phytagel at pH 5.7. Seeds were vernalized for 3 days at 4 °C before being transferred to an incubation chamber for two weeks. Seedlings were then transplanted into Sunshine Mix #1 soil and kept at 22 °C a 16-h photoperiod. For *vitamin c defective 2* (SALK_146824) Arabidopsis plants, T-DNA insertion was verified with PCR. One rosette leaf of four-week old Arabidopsis plants was inoculated with a 4.5mm diameter agar plug containing growing *S. sclerotiorum* hyphae under high humidity levels maintained with a large clear plastic bag. Progression of infection at 24 and 48hpi was determined by measuring the leaf length and width and calculating elliptical area of infection in at least 14 plants per treatment. Progression of infection at 72hpi was determined by dividing total number of infected rosette leaves by total number of inoculated rosette leaves. RNA isolation, cDNA synthesis, and qPCR reactions were carried out as described above. Quantification of ascorbic acid (ASC) levels was performed as per Gillespie and Ainsworth (2007).

### Accession numbers

Raw and processed sequence data are available from the National Center for Biotechnology’s (NCBI) Gene Expression Omnibus as GEO Series GSE81545.

## Results

### Petal inoculation is essential for *S. sclerotiorum* infection

**Fig. 1.**
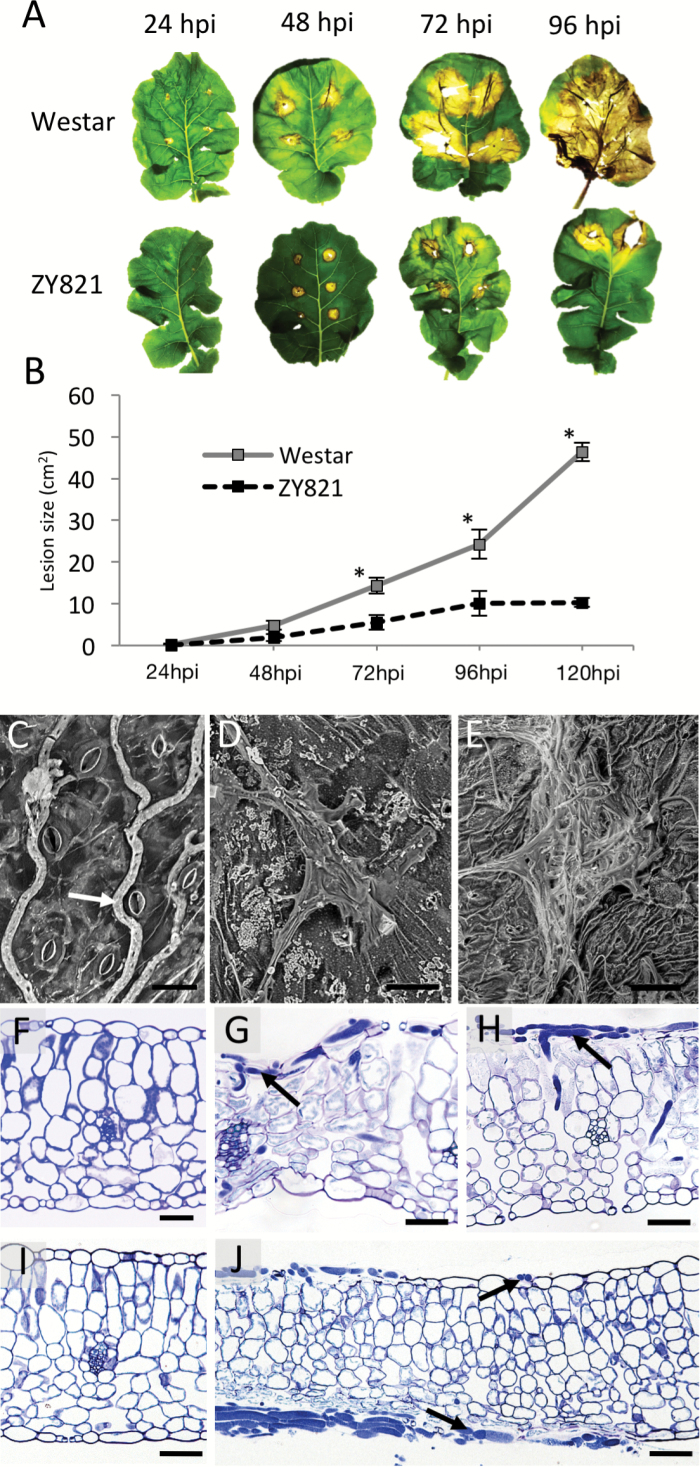
Characterization of the *Brassica napus*-*Sclerotinia sclerotiorum* pathosystem. (A) *S. sclerotiorum* lesion progression in *B. napus* susceptible (cv. Westar) and tolerant (cv. ZY821) genotypes. (B) Lesion size measurements±standard deviation from 24–120 hours post-inoculation (hpi) in Westar and ZY821, * indicates ANOVA *P*-value<0.05. (C) Scanning electron micrograph of *S. sclerotiorum* hyphae (arrow) avoiding stomata on the adaxial epidermis of Westar 24hpi. Representative *S. sclerotiorum* infection cushions on abaxial epidermis of (D) Westar and (E) ZY821. (F-J) Light microscopy of transverse leaf sections stained with Toluidine Blue O. (F) Un-inoculated section of healthy Westar tissue. (G) Westar leaf 48hpi showing extensive membrane shrinkage caused by *S. sclerotiorum*, and darkly staining hyphae (arrow). (H) Infection cushion (arrow) on adaxial epidermis of Westar 48hpi. (I) Un-inoculated healthy section of ZY821 tissues. (J) Cross-section of infected ZY821 leaves showing limited cellular degradation in advance of *S. sclerotiorum* hyphae (arrows). Scale bars, C: 80µm; D: 60µm; E: 120µm; F-J: 50µm.

We developed a *S. sclerotiorum* petal inoculation method to identify how *B. napus* responds to fungal attack at the earliest stage of the infection process. Disease progression was assayed in universally susceptible (cv. Westar) and tolerant (cv. ZY821) genotypes of *B. napus.* The petal inoculation method replicates the infection process that naturally occurs in the field (Jamaux *et al.*, 1995; Huang *et al.* 2008). When leaves were infected with a senescing petal, we observed 100% infection compared to 20% with a fresh petal. Profound differences in disease progression were observed at 48hpi, with little green tissue remaining by 96hpi in the susceptible host (Fig. 1A). We measured lesion area and observed statistically larger lesions in susceptible hosts at 72, 96, and 120hpi, with a 78% increase by 120hpi (Fig. 1B). Scanning electron microscopy revealed topographic features of the infection process directly at the infection site. *S. sclerotiorum* hyphae did not appear to penetrate host tissues via stomata (Fig. 1C). Infection cushions were observed in both cultivars (Fig. 1D and 1E); however, they were qualitatively larger and more abundant on the ZY821 leaf surface.

We then used light microscopy directly at the infection site in the susceptible (Fig. 1F-H) and tolerant (Fig. 1I-J) hosts. Data revealed *S. sclerotiorum* growth inside susceptible epidermal cells at 48hpi and membrane shrinkage in advance of the infecting hyphae (Fig. 1G). Cellular degradation indicated by membrane shrinkage was only observed in the cells surrounding the hyphae in the tolerant cultivar. Similarly, fungal hyphae could penetrate the mesophyll in susceptible leaf tissues (Fig. 1H) more readily than in tolerant hosts (Fig. 1J).

### Global patterns of gene activity in response to *S. sclerotiorum*

**Fig. 2.**
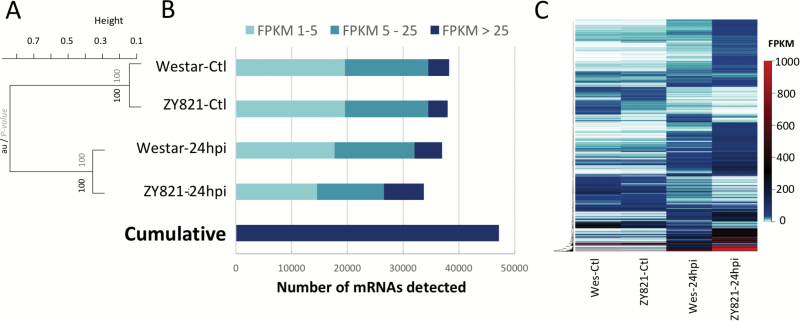
RNA Sequencing of susceptible (Westar) and tolerant (ZY821) genotypes of *B. napus* infected with *S. sclerotiorum.* (A) Hierarchical clustering of genes detected with a minimum FPKM of 1, with approximately unbiased (au) values in black and bootstrap P-values in grey. (B) Distribution of transcript abundances in FPKM normalized across the four treatments. (C) Clustered heatmap of top 10,000 most highly abundant mRNAs.

To interrogate global changes in gene activity and elucidate potential regulatory elements contributing to the tolerant phenotype observed in ZY821, we performed high-throughput RNA-seq on both genotypes at 24hpi using ascospore- and mock-inoculated petals (Fig. 2). Reads were mapped to the reference assembly of *B. napus* v5.0 (Chalhoub *et al.*, 2014) and *S. sclerotiorum* (Amselem *et al.*, 2011). Total numbers of reads per biological replicate for each species are presented in Tables S1 and S2 respectively. For plant transcripts, we considered a transcript ‘detected’ if the Fragments Per Kilobase of gene per Million reads mapped (FPKM) level was≥1 (Bhardwaj *et al.*, 2015; Chan *et al.*, 2016). Hierarchical clustering of detected transcripts grouped samples together based on treatment, indicating both cultivars underwent a broad shift in gene activity in response to *S. sclerotiorum* infection (Fig. 2A). Transcripts were further divided into those with low (1≤FPKM<5), moderate (5≤FPKM<25), or high (FPKM≥25) accumulation levels. Mock-inoculated samples in both cultivars had a similar distribution of lowly, moderately, and highly accu mulating mRNAs (Fig. 2B). In these mock treatments, a total of 38,254 transcripts were detected in Westar and 37,977 in ZY821. In leaf tissues infected with *S. sclerotiorum*, we detected 36,986 mRNAs in the susceptible host and 9% fewer (33,720) in ZY821. In the tolerant host, a greater percentage of detected transcripts had an FPKM>25 (21.1%) as compared to susceptible Westar (13.4%). Inclusive of all treatment groups we detected 47,154 mRNAs, with the most abundant 10,000 mRNAs showing distinct accumulation patterns between the two cultivars and treatments (Fig. 2C).

### Identification of unannotated genes with roles in plant defense

To identify whether previously unannotated genes play a role in *B. napus* defense to *S. sclerotiorum*, reads of individual samples were assembled, merged, and compared with the existing *B. napus* transcriptome annotation (Table S1, Chalhoub *et al.*, 2014; Trapnell *et al.*, 2012). To strengthen predictive power, only detected transcripts outside of existing gene models with protein blast hits to *A. thaliana* were retained. Using these criteria, a total of 1,233 previously unannotated transcripts were identified (Table S3) and graphed as a heatmap (Fig.S1). The 20 most abundant novel transcripts are presented in Table S4, and include potential homologues of characterized Arabidopsis lipid transfer genes, sulfur assimilators (*ATP SULFURYLASE 1*), protein regulators (*POLYUBIQUITIN 10*), and signal transducers *(MAP KINASE 4*; *MPK4*).

### Differential gene activity in response to *S. sclerotiorum* infection

**Fig. 3.**
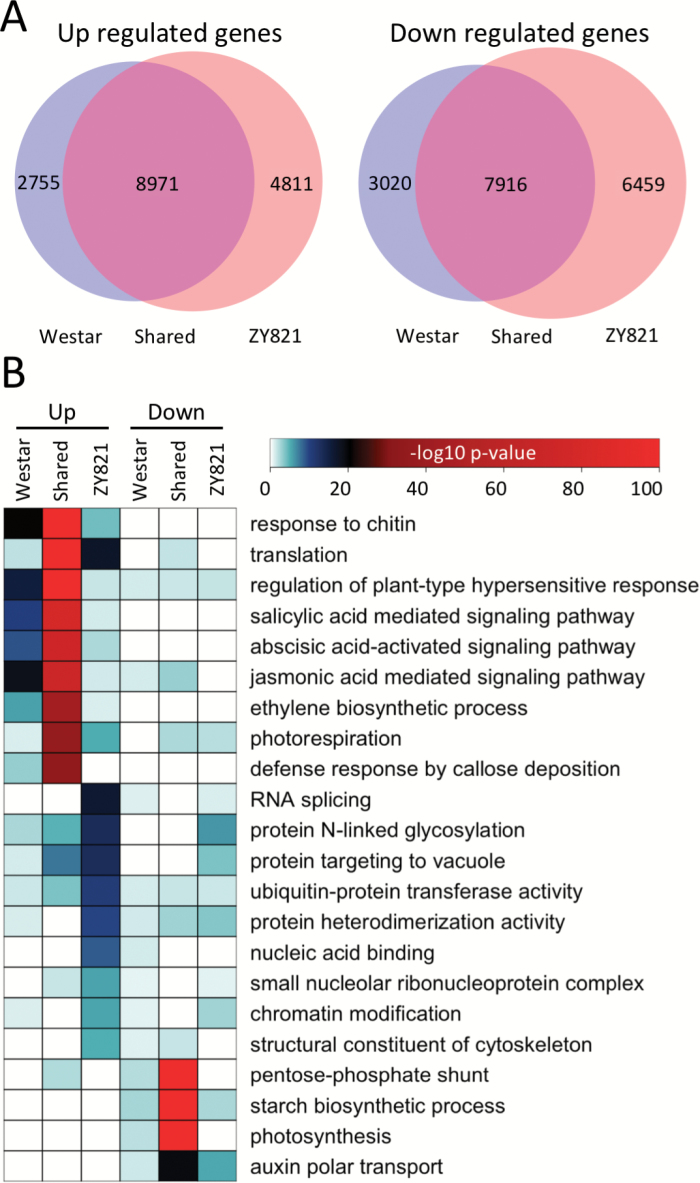
Differential gene expression and gene ontology (GO) analysis. (A) Euler diagrams showing number of genes differentially expressed in susceptible (Westar) and tolerant (ZY821) genotypes of *B. napus* infected with *S. sclerotiorum* (false discovery rate<0.05). Overlapping segments represent genes differentially expressed in both genotypes. (B) Heatmap of a subset of significantly enriched GO terms. GO terms are considered statistically significant if the hypergeometric *P*-value<0.001. See also Table S5.

We then identified gene sets activated in *B. napus* in response to *S. sclerotiorum* in both susceptible and resistant cultivars. We found 53% of genes detected were differentially expressed (false discovery rate<0.05) in susceptible leaves with a total of 11,726 genes being differentially up- and 10,936 differentially downregulated. Overall, 65% of the genes were differentially expressed in the tolerant cultivar, with 13,782 up- and 14,375 downregulated. Although the majority of differentially expressed genes were shared between the two cultivars, we found relatively large number of genes specifically differentially expressed in each cultivar (Fig. 3A). This suggests large coordinated shifts in gene expression in susceptible and resistant hosts at early stages of the infection process.

### Gene ontology analysis reveals biological processes shared between tolerant and susceptible *B. napus* plants responding to *S. sclerotiorum*

To query the biological processes encoded within differentially expressed gene sets, we used the GO enrichment function of SeqEnrich (Becker *et al.*, 2017b). GO terms were considered significantly enriched if the hypergeometric *P*-value<0.001 (Fig. 3B, Table S5). We predicted plant defense hormones to be active in both susceptible and tolerant leaves in response to *S. sclerotiorum*. Salicylic acid (*P*=2.72E-79), abscisic acid (*P*=1.85E-73), and jasmonic acid (*P*=2.73E-71) signalling processes, as well as response to ethylene (*P*=1.89E-69), were all enriched in genes upregulated in response to the fungus in both treatments (Table S5).

In response *S. sclerotiorum*, both susceptible and resistant hosts activated genes enriched for photorespiration (*P*=2.67E-36) and downregulated genes enriched for photosynthesis (*P*=5.06E-137) and starch biosynthetic processes (*P*=1.27E-180), suggesting inhibition of growth-related bioprocesses in response to pathogen. Likewise, in both genotypes shared downregulated genes were associated with auxin-activated signaling pathways (*P*=3.27E-06) and auxin polar transport (*P*=4.69E-06). Together, this is indicative of large-scale transcriptional reprogramming following infection with *S. sclerotiorum* in both genotypes that may modulate growth and metabolism.

### Identification of biological processes activated specifically in *B. napus* tolerant to *S. sclerotiorum*

Next, we were interested in exploring the biological processes active in the 4,811 genes upregulated exclusively in tolerant hosts following pathogen exposure (Fig. 3B). We identified bioprocesses previously unassociated with *B. napus* defense against *S. sclerotiorum*, including RNA-splicing (*P*=3.49E-17) and chromatin modification (*P*=3.43E-06). Additionally, this gene set was associated with GO terms related to protein sorting and trafficking, including protein N-linked glycosylation (*P*=4.26E-14) and protein targeting to vacuole (*P*=5.52E-14), in which *VACUOLAR SORTING RECEPTOR 1*, vSNARE protein family members, and *BZIP53* transcripts were also differentially up-regulated. Interestingly, the data also point to a role of the structural component of the cytoskeleton (*P*=8.60E-06), with both *ACTIN 2* (*ACT*2) and *TUBULIN BETA CHAIN 4* upregulated in ZY821 (Fig. S4, Table S6).

**Fig. 4.**
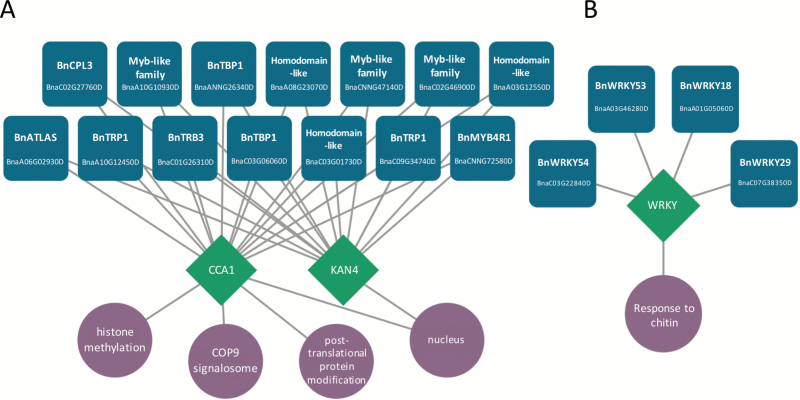
Putative transcriptional regulation of *Sclerotinia* tolerance in *B. napus*. (A) Putative circuit controlled by CIRCADIAN CLOCK ASSOCIATED 1 (CCA1) and KAN4 binding sites. Transcription factors (blue squircles) are predicted to bind to the over-represented (*P*<0.001) DNA motifs (green diamonds) to control GO terms (purple circles). (B) Putative transcriptional circuit regulated by WRKY DNA binding motif.

To identify potential regulatory networks controlling these biological processes, we used SeqEnrich (Becker *et al.*, 2017b), which contains a curated database containing all current information on *B. napus* TFs, DNA sequence motifs, and GO terms. Using this program, TFs were associated with DNA sequence motifs overrepresented (*P<*0.001) in gene promoters within enriched GO terms. Two networks were identified in genes specifically upregulated in tolerant hosts at 24hpi (Table S7). C-terminal domain phosphatase-like 3 (BnaC02G27760D) and MYB TFs were predicted to bind to the CCA1 and KAN DNA promoter motifs upstream of genes enriched for histone methylation and protein modification (Fig. 4A). The same analysis also identified four WRKY DNA-binding proteins (WRKYs) predicted to contribute to the plant response to chitin (*P*=3.44E-05, Fig. 4B). The association of the CCA1 DNA motif with genes specifically upregulated in the tolerant cultivar led us to investigate whether circadian rhythm-regulated genes were globally affected at the RNA level in the two cultivars following exposure to *S. sclerotiorum*. We plotted the corresponding FPKMs of homologous genes shown to be circadian regulated in Arabidopsis (Covington *et al.*, 2008), and show clear differences in how circadian clock associated gene sets respond to *S. sclerotiorum* in the two *B. napus* genotypes (Fig. S2). Both homologues of the master clock regulator *CCA1* shared similar expression profiles in both genotypes, however *LHY* homologues accumulated at an average of 4.5 times higher in ZY821 control samples (Table S6).

### Coexpression analysis reveals distinct patterns of gene activity and regulatory networks between susceptible and tolerant genotypes

**Fig. 5.**
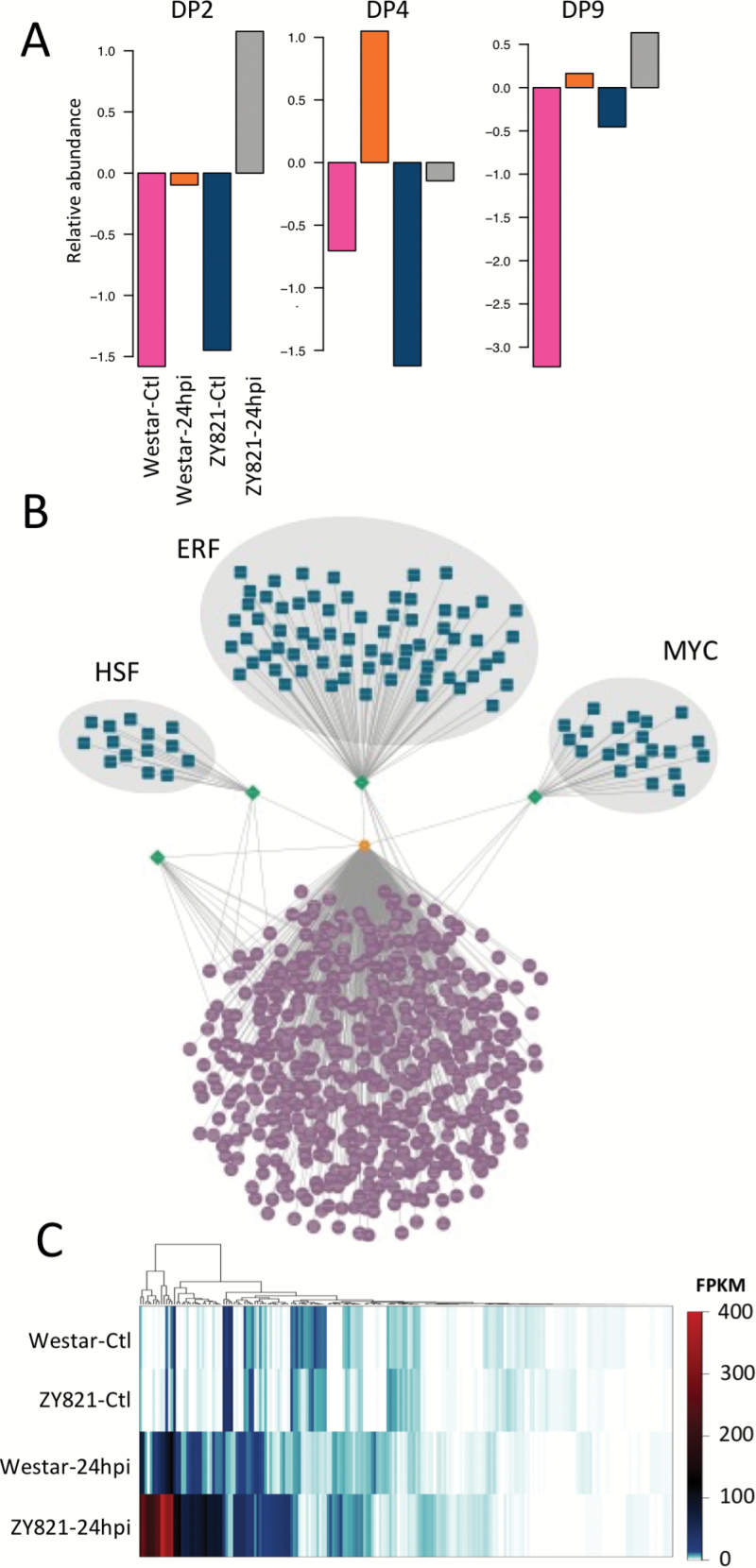
Dominant patterns (DPs) of gene activity in susceptible (Westar) and tolerant (ZY821) genotypes of *B. napus* infected with *S. sclerotiorum* using fuzzy k-means clustering and enrichment analysis. (A) Bar plots representing relative abundance of mRNAs assigned to three DPs. (B) Predicted transcriptional module identified in DP2 (gold hexagon) accumulating primarily in the tolerant *B. napus* genotype at 24hpi. Sets of Heatshock Factors (HSF), Ethylene Response Factors (ERF) and MYC transcription factors (blue squircles) are predicted to bind to their overrepresented DNA motifs (*P*<0.001) up stream of genes enriched for gene ontology terms (purple circles). (C) Heatmap of all putative ERF coding transcripts.

We used a modified fuzzy *K*-means clustering algorithm to identify coexpressed transcripts with finer patterns of gene activity than detectable with differential expression, and to group genes of similar expression profiles (Belmonte *et al.*, 2013). Ten dominant patterns of gene activity (DPs) were determined, each containing anywhere from 599 to 9,536 genes with a median of 1,284 (Table S8). Our analysis identified patterns containing mRNAs abundant in infected Westar (DP4), infected ZY821 (DP2), or in both genotypes during infection (DP9; Fig. 5A). We also resolved patterns containing mRNAs that accumulated based on genotype. For example, mRNAs of DP8 accumulated primarily in Westar, and DP3 mRNAs primarily in ZY821 (Fig. S3).

Predicted TF-promoter interactions identified a putative transcriptional circuit in mRNAs accumulating in tolerant leaves at 24hpi (DP2). Heat-shock transcription factors (HSPs), ethylene responsive element binding factors (ERFs), and MYC-family TFs (MYCs) were predicted to control biological processes including defense signaling, protein biosynthesis and trafficking, secondary metabolite production, and redox regulation (Fig. 5B, Table S7). Further investigation of ERF-coding transcripts showed that the majority were upregulated in both genotypes following infection, but accumulated to much higher levels in the tolerant host (Fig. 5C).

### Activation of the cellular redox system in tolerant *B. napus* leaves

**Fig. 6.**
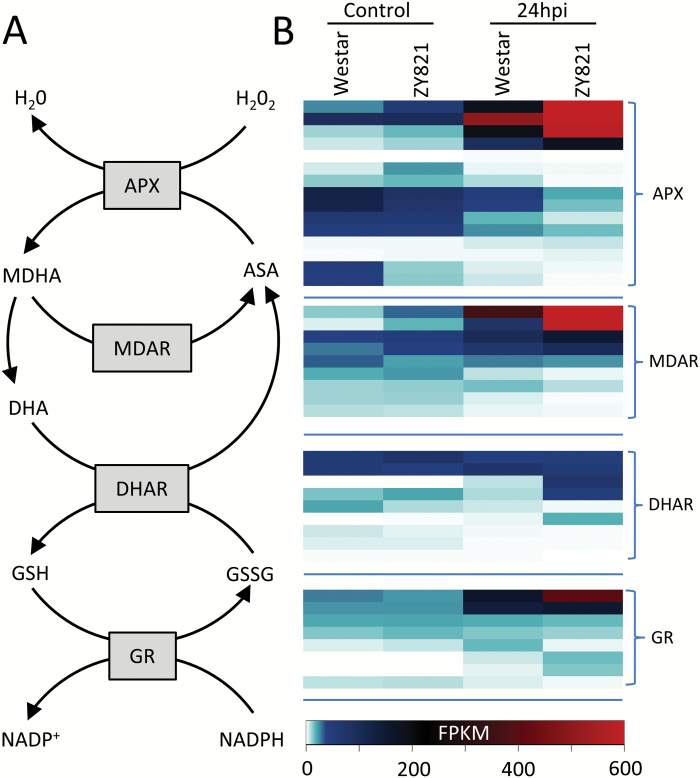
Transcript analysis of ascorbate-glutathione redox cycling pathway in susceptible (Westar) and tolerant (ZY821) genotypes of *B. napus* infected with *S. sclerotiorum*. (A) Overview of the ascorbate-glutathione redox enzymes and products. (B) Accumulation of transcripts from all homologues of enzymes involved in pathway represented as heatmap.

Identification of enriched redox-related GO terms in DP2 (Table S5) led us to investigate differences in the ascorbate-glutathione redox cycle between the two genotypes in response to pathogen attack (Fig. 6A). Transcripts encoding homologues of integral enzymes in the ascorbate-glutathione pathway including cytosolic *ASCORBATE PEROXIDASE* (*APX*) and *GLUTATHIONE REDUCTASE* (*GR*) accumulated up to 15- and 33-fold higher respectively, in infected leaves of tolerant ZY821 than in Westar (Fig. 6B).

**Fig. 7.**
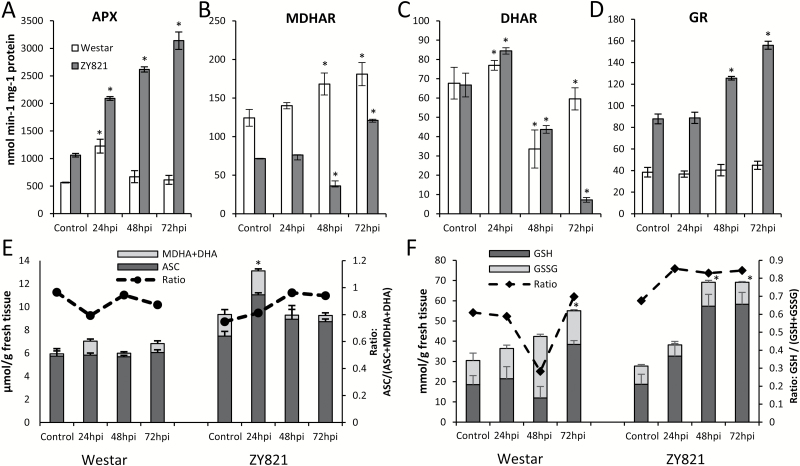
Physiological measurements of ascorbate-glutathione redox cycling pathway in susceptible (Westar) and tolerant (ZY821) genotypes of *B. napus* infected with *S. sclerotiorum*. (A) Ascorbate peroxidase, (B) monodehydroascorbate reductase, (C) dehydroascorbate reductase, and (D) glutathione reductase enzymatic activity measured from infected leaves of *B. napus*. Levels and ratios of reduced and oxidized (E) ascorbate and (F) glutathione. Data represent mean of biological replicates±standard deviation, statistically significant change from control levels indicated by *. Abbreviations: APX, ascorbate peroxidase; ASC, reduced ascorbate; DHA, dehydroascorbate; DHAR, dehydroascorbate reductase; GR, glutathione reductase; GSH, reduced glutathione; GSSG, oxidized glutathione; MDHA, monodehydroascorbate; MDHAR, monodehydroascorbate reductase; NADP, nicotinamide adenine dinucleotide phosphate oxidized form; NADPH, nicotinamide adenine dinucleotide phosphate reduced form.

To study whether elevated transcript levels corresponded to real changes in ascorbate-glutathione redox balance we measured enzyme activities by quantifying small-molecule substrates (Fig. 7). APX activity was between 41–80% higher across each time point in ZY821 compared to Westar (Fig. 7A). Additionally, GR activity doubled in tolerant plants by 72hpi, whereas there was no significant increase in susceptible hosts (Fig. 7D). The total cellular ascorbate pool in ZY821 was higher than Westar for all time points, with only the ZY821 24hpi treatment showing a significant deviation from the endogenous levels in the control (Fig. 7E). Total glutathione levels and the glutathione redox ratio (GSH/(GSH+GSSG)) were similar in controls of both genotypes. In ZY821, the glutathione redox ratio remained relatively constant following infection and the total glutathione pool increased 60% at 48hpi. In Westar, the glutathione redox ratio decreased from 0.6 to 0.3 over the same period and the total glutathione pool increased by only 28% at 48hpi (Fig. 7F).

### Redox homeostasis mutants in Arabidopsis are hyper-susceptible to *Sclerotinia*

**Fig. 8.**
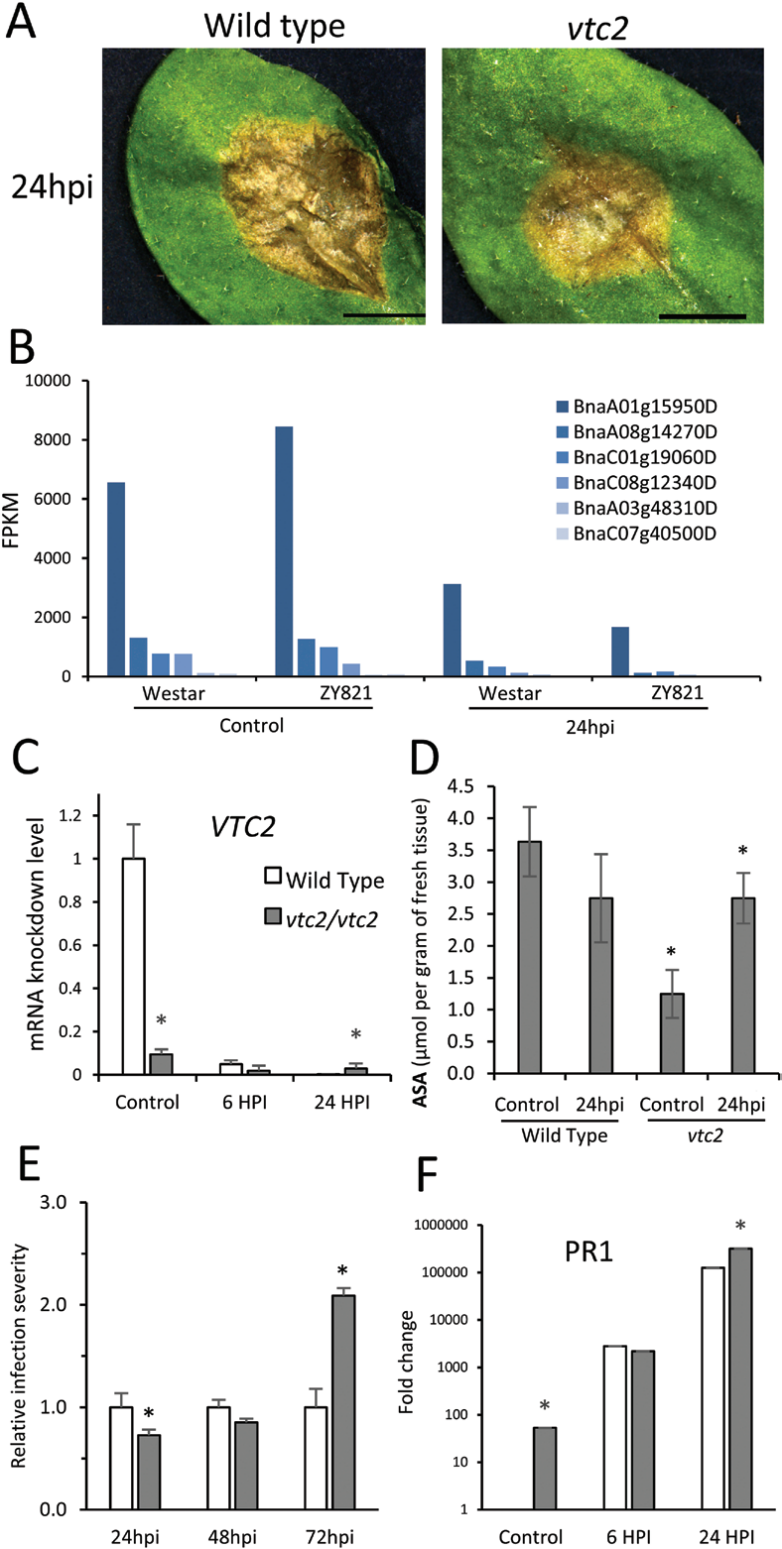
Redox control of *Sclerotinia* infection in Arabiodpsis (A) Wild-type Col and *vtc2* mutants infected with Sclerotinia 24hpi(B) FPKM levels of all detected homologues of *VTC2* in *S. sclerotiorum* infected leaves of *B. napus*. (C) Relative mRNA levels of *VTC2* at 6 and 24hpi in infected leaves measured by qPCR. (D) Ascorbate levels in wild-type and *vtc2* Arabidopsis. (E) Lesion progression measured 72hpi compared to WT control (F) qPCR relative mRNA levels of infected plants for *PATHOGENESIS-RELATED GENE 1* (*PR1*). Data represent mean of biological replicates±standard error, statistical significance was determined using a Students t-test with a minimum *P*<0.05 for statistical significance.

To further validate the role of redox homeostasis in the plant defense response to *S. sclerotiorum,* we studied an Arabidopsis knockdown line of the rate-limiting enzyme of ascorbate biosynthesis, *vitamin c defective 2* (*vtc2*, Fig. 8A). Loss-of-function mutants produce 10–25% of wild-type ascorbate levels (ASC, Pavet *et al.*, 2005), limiting their capacity to buffer redox stress through the ascorbate-glutathione cycle. In *B. napus*, all *VTC2* homologues are downregulated in response to infection at 24hpi (Fig. 8B), which corresponds to our observations in Arabidopsis as measured by qPCR (Fig. 8C). We assayed ASC levels at 24hpi in wild-type and *vtc2* Arabidopsis plants. Although we found a 70% reduction of ASC in mock-inoculated *vtc2*, we observed a rescue to wild-type levels during infection (Fig. 8D). Phenotypically, infected *vtc2* plants initially showed a delayed lesion spread; however, at 72hpi, the infection is twice as severe (Fig. 8E). We assessed activation of the plant defense response via qPCR detection of marker *PATHOGENESIS-RELATED 1* (*PR1*; Fig. 8F) and found higher baseline levels in *vtc2* plants, with increased abundances in both lines following contact with the pathogen.

## Discussion

Our analysis of the *B. napus*-*S. sclerotiorum* interaction directly at the host pathogen interface provides novel insight into the structural, molecular, and physiological changes in the *B. napus* leaf at the earliest stages of the infection process. We identified putative transcription factor circuits controlling biological processes and defense genes within the host plant in response to *S. sclerotiorum* controlling lesion spread and disease progression. A comprehensive investigation of redox homeostasis following *S. sclerotiorum* infection revealed pronounced genotypic differences between our two experimental host plants, providing physiological evidence for the tolerant phenotype of ZY821.

Previous studies investigating this pathosystem towards the later stages of the infection process in stem tissues used artificial nutrient sources to promote infection and is not reflective of disease transmission in the field (Wu *et al.*, 2016; Zhao *et al.*, 2009). Our method of using senescing petals simulates field conditions, as senescing petals likely provide the nutrients required for *Sclerotinia* to finance the production of toxins, cell wall degrading enzymes, and appressoria required to penetrate host tissues (Bolton, 2006). Interestingly, we did not observe stomatal penetration of leaves in either genotype, even though stomata are directly regulated via OA (Stotz and Guimaraes, 2004) and serve as ideal entry points for other pathogenic fungi (Kim *et al.*, 2011). Instead, SEM and light microscopy data revealed advancing hyphae penetrating the physical barriers of the plant via infection cushions, the occurrence of which is correlated to *Sclerotinia* pathogenicity (Jurick and Rollins, 2007). An abundance of complex appressoria on the epidermis of ZY821 suggests *S. sclerotiorum* requires additional resources for colonization of the tolerant host. The cellular differences observed between the susceptible and tolerant cultivars provide additional structural evidence for the colonization strategy of *S. sclerotiorum* on the leaf surface.

Global RNA profiling of the *B. napus* infection site within the first 24 hours of the host pathogen interaction revealed large and coordinated shifts in gene activity. While large numbers of differentially expressed genes have been reported in stem tissues of *Sclerotinia*-resistant plants (Wu *et al.*, 2016), we show a transcriptional response in ZY821 within hours of infection that may limit *S. sclerotiorum* penetration and colonization with the leaf. We discovered many differences in the amplitudes and attenuations of genes fundamental to the defense process, providing a new understanding of the molecular foundations of tolerance to *Sclerotinia*. For example, activation of both SA and JA/ethylene signal transduction networks in leaves 24 hpi supports a conserved defense response in both stem and leaf tissues following *Sclerotinia* infection (Wu *et al.*, 2016; Zhao *et al.*, 2009). Interestingly, transcripts for SA biosynthesis, including *ISOCHORISMATE SYNTHASE 1* (*ICS1*) homologues accumulated up to six times more in the susceptible line than in ZY821. This contradicts previous work of Zhao *et al.* (2009) who found *ICS1* homologues downregulated in stem tissues of the same two genotypes 24hpi using a *B. napus* microarray. Although these differences may be explained by differential activation of homologues not included in the array used by Zhao *et al.* (2009), however, Wu *et al.* (2016) also found downregulation of *ICS1* levels in both susceptible and resistant stem tissues. Thus, our work suggests organ-specific transcriptional regulation of hormone biosynthesis in response to *S. sclerotiorum*.

Further, mRNAs associated with ethylene biosynthesis and signaling were more abundant in tolerant leaves. Levels of *OCTADECANOID-RESPONSIVE ARABIDOPSIS 59* (*ORA59*), a key transcriptional activator of ethylene and JA signaling (Caarls *et al.*, 2015), were 6.5-fold higher in tolerant hosts. Given that SA directly represses *ORA59* transcription (Caarls *et al.*, 2015), accumulation of SA through ICS1 in susceptible host leaves may be responsible for limiting JA/ethylene associated defence activation in foliar tissues. This suggests that upstream restriction of SA biosynthesis may prevent SA-mediated JA antagonism (Van der Does *et al.*, 2013) in tolerant *B. napus* leaves and highlights the importance of studying the plant response at the first point of contact with *S. sclerotiorum*.

MAPK signalling cascades are essential to defense and transduce JA signals to transcriptional reprogramming (Meng and Zhang, 2013; Takahashi *et al.*, 2007). Our study identified differentially expressed MAPK genes between both genotypes, suggesting differences in how cells interpret and respond to biotic stressors. In Arabidopsis, MPK4 is a important regulator of PTI and activator of WRKY33 (Qiu *et al.*, 2008; Zhang *et al.*, 2012). We identified an unannotated MPK4 homologue (*CUFF.20400.1*) detected exclusively and abundantly in infected ZY821 through our transcriptional analysis, and validated using qPCR. Thus, CUFF.20400.1-mediated signaling may contribute to *S. sclerotiorum* tolerance in ZY821 additionally. This also illustrates how RNA sequencing is an effective tool to identify critical genetic differences in cultivar-specific defense transcripts beyond existing genome assemblies.

Suppression of fungal attack relies on timely activation of TFs controlling defense gene networks. We identified a suite of WRKY TFs putatively controlling the plant response to chitin within 24hpi in tolerant hosts. Specifically, WRKY18, which contributes to Arabidopsis defense against the necrotroph *Botrytis cinerea* (Xu *et al.*, 2006), was identified in our TF network. This suggests that WRKY18, along with additional WRKY TFs including WRKY 29, 53 and 54, may have evolved to play a regulatory role in mitigating attack from necrotrophic fungi. Thus, our predictive transcription factor network activated specifically in the tolerant cultivar provides a platform for future studies to uncover the role of WRKY TFs as master regulators of necrotrophic defense in *B. napus* and other crop species.

Following infection of tolerant *B. napus*, our data predict development of complex TF-DNA motif interactions that include ERF and MYC TFs shown to be downstream of JA signalling (Lorenzo *et al.*, 2004; Niu *et al.*, 2011). Among these TFs are homologues of *MYC3*, a bHLH TF that interacts with MYBs to activate glucosinolate biosynthesis in Arabidopsis (Schweizer *et al.*, 2013). All three homologues of *INDOLE GLUCOSINOLATE METHYLTRANSFERASE 5* detected in this study (*BnaA07G33060D, BnaC06G37610D*, and *BnaC06G21620D*) are co-expressed and predicted to be controlled through network interactions, offering novel insight into how glucosinolate production is transcriptionally controlled in response to *S. sclerotiorum*. Although the interactions remain putative, our network analyses provide an elegant and direct avenue for further investigation using functional characterization pipelines.

Global transcriptional reprogramming observed in tolerant leaf tissues, which expressed fewer genes following infection than the susceptible line provides evidence for genome-wide control of the genetic regulatory machinery following interaction with *Sclerotinia*. Enrichment of genes associated with DNA methylation controlled by *TELEMORIC BINDING PROTEIN* homologues in genes specifically expressed in ZY821, and higher accumulation levels of *SET* homologues hint at differences in how the two cultivars adjust chromatin and chemical modifications of genetic material in response to fungal infection. Since DNA methylation is essential to plant defense (Dowen *et al.*, 2012), and transcriptional regulation of JA/ET genes requires the histone methyltransferase *SET DOMAIN GROUP8* in Arabidopsis (Berr *et al.*, 2010), epigenetic control mechanisms may directly contribute to tolerance to *S. sclerotiorum*.

Reactive oxygen species (ROS) produced through the PTI pathway serve as important defense signalling molecules, but can also damage the host’s molecular machinery causing cell death (Scheler *et al.*, 2013; Tripathy and Oelmüller, 2012). Within 24 hours of infection, at the leaf-fungal interface, we observed elevated redox buffering capacity in tolerant leaf tissues through genes contributing to oxidative stress and glutathione metabolic processes. Enzyme activity of APX and GR further validated gene expression data through the qualification of their small molecule substrates, ASC and GSH - critical components of the plant immune system (Foyer and Noctor, 2011; Ishikawa and Shigeoka, 2008). Interestingly, increased ASC at 24hpi in tolerant leaves does not correspond to transcript levels of VTC2, the rate-limiting enzyme in ASC biosynthesis. All *VTC2* homologues were downregulated during infection in both genotypes, however transcript levels of *ERF98*, a positive regulator of ASC biosynthesis (Wang *et al.*, 2013; Zhang *et al.*, 2012), were considerably higher in tolerant leaf tissues. These data further support our transcriptional circuit that places an *ERF98* homologue, *BnaA07G06750D,* as a regulator of redox related defense processes, and provides novel insight into the transcriptional regulation of the complex networks underlying tolerance to *S. sclerotiorum*.

Activation of the ASC-GSH pathway in tolerant leaves of ZY821 likely contributes to the lack of cellular degradation observed in advance of fungal hyphae, thus limiting nutrient availability from OA-induced cell death (Kim *et al.*, 2008). The importance of redox regulation and homeostasis in defense against *S. sclerotiorum* is further validated by the hyper-susceptible phenotype of *vtc2* Arabidopsis. Delayed lesion growth in *vtc2* plants at early infection stages is likely due to defense priming caused in part by the activation and nuclear localization of redox sensitive Nonexpressor of PR Genes 1 (NPR1), which transcriptionally activates *PR1* and other defense regulators (Tada *et al.*, 2008; Pavet *et al.*, 2005). The *vtc2* Arabidopsis plants constitutively express *PR1* among other antimicrobial genes and accumulate phytoalexins at higher levels than wild-type plants (Colville *et al.*, 2008), amounting to elevated defense capacities at the initial contact with the pathogen. Although this offers protection initially, inability to appropriately respond to *S. sclerotiorum* and buffer OA-induced ROS compromises defense over time, as seen in *vtc2* plants infected with necrotrophic fungal pathogen *Alternaria brassicola* (Botanga *et al.*, 2012). Taken together, we show that the coordination and functioning of cellular redox buffering systems at the host-pathogen interface has a profound role in the defense response against *S. sclerotiorum*, providing a physiological mechanism responsible for management of tissue damage and the tolerant phenotype of ZY821.

The current study presents a timely investigation into the transcriptional and physiological changes contributing to *S. sclerotiorum* tolerance in the ZY821 genotype directly at the site of infection at the host-pathogen interface of the leaf. While we have yet to fully understand the molecular underpinnings of *S. sclerotiorum* infection severity in *B. napu*s or the full spectrum of genes required for genetic resistance, we have uncovered new molecular and physiological processes associated with tolerance at the first point of infection under real-world conditions. The wholesale transcriptional reprogramming undergone by plant cells infected with fungal pathogens is an intricately regulated process controlled through the activity of transcriptional circuits. This regulatory analysis highlighted the transcriptional control of redox response and highlights the critical nature of redox homeostasis for *B. napus* tolerance to *S. sclerotiorum*.

## SUPPORTING INFORMATION

Supplementary data are available at *JXB* online.

Fig. S1. Clustered heatmap of all 1233 newly identified genes based on FPKM levels

Fig. S2. Heatmap of transcript levels in FPKM of all homologues of genes identified as circadian regulated in Arabidopsis (Covington *et al.*, 2008).

Fig. S3. Dominant patterns of gene activity discovered using fuzzy k-means clustering analysis. Bar plots represent relative accumulation level of transcripts belonging to each pattern.

Fig. S4. Enzymatic and ascorbic acid (ASC) analysis. Enzyme activity levels of (A) monodehydroascorbate reductase (MDAR) and (B) dehydroascorbate reductase (DHAR) from healthy and infected leaf tissues. (C) Levels of reduced ascorbate (ASC) and oxidized oxidized forms monodehyrdoascorbate (MDHA) and dehydroascorbate (DHA), and ASC redox ratio.

Fig. S5. Quantitative reverse transcription PCR of select genes. Relative fold changes from RNA-sequencing data are displayed as bars with qPCR levels as dots.

qPCR of selected transcripts

Table S1. Number of Illumina sequence reads that map to the *Brassica napus* genome.

Table S2. Number of Illumina sequence reads that map to the *Sclerotinia sclerotiorum* genome.

Table S3. Gene annotation and FPKM levels.

Table S4. Top 20 most highly abundant transcripts uncovered in the novel transcript discovery analysis.

Table S5. Gene Ontology summary.

Table S6. Transcript levels in FPKM of selected genes.

Table S7. Module tables.

Table S8. Fuzzy K means dominant patterns gene lists.

Table S9. Primer sequences used for quantitative reverse transcription PCR and genotyping of Arabidopsis mutants.

## Supplementary Material

supplementary-Table-S1-S2,S4,S6,S9

supplementary-Table-S3

supplementary-Table-S5

supplementary-Table-S7

supplementary-Table-S8

Supplementary-Figures-S1-S4
