# Supplementary material for: RNA sequencing of Brassica napus reveals cellular redox control of Sclerotinia infection
Source: J Exp Bot. 2017 Sep 27;68(18):5079–91. doi: 10.1093/jxb/erx338 (PMC5853404; doi:10.1093/jxb/erx338)
Supplement: supplementary-Table-S1-S2,S4,S6,S9 [file erx338_suppl_supplementary-table-s1-s2,s4,s6,s9.pdf]

Table S1 Number of Illumina sequence reads that map to the *Brassica napus* genome v5.0.

Cultivar & Treatment	Replicate	Trimmed reads (millions)	Mapped reads (millions)	overall mapping %	% uniquely mapping
Westar Control	1	12.4	9.4	75.5	83.6
	2	13.2	10.4	79	82.8
	3	33.1	26.3	79.3	83.3
Westar 24hpi	1	34.1	21.3	62.5	83.5
	2	35.6	22.6	63.5	84.3
	3	44.0	29.9	68	83
ZY821 Control	1	48.2	38.1	79.1	82.6
	2	2.4	1.9	79.1	83.0
	3	30.4	24.8	81.4	83.9
ZY821 24hpi	1	19.7	12.6	63.7	84.1
	2	30.4	18.4	60.6	84.0
	3	43.0	28.4	66.1	83.9

Table S2 - Number of Illumina sequence reads that map to the *Sclerotinia sclerotiorum* genome v2.

Cultivar & Treatment	Replicate	Trimmed reads (millions)	Mapped reads (millions)	overall mapping %	% uniquely mapping
Westar Control	1	12.3	0.0	0.0	73.9
	2	13.2	0.0	0.1	91.1
	3	33.1	0.0	0.0	92.8
Westar 24hpi	1	40.3	7.8	19.4	99.1
	2	37.0	6.5	17.5	99.0
	3	52.0	8.1	15.6	99.0
ZY821 Control	1	48.2	0.0	0.0	84.5
	2	2.4	0.0	0.0	56.9
	3	30.4	0.0	0.1	86.4
ZY821 24hpi	1	31.5	6.3	20.0	98.9
	2	44.4	6.7	15.1	98.4
	3	20.5	3.1	15.0	98.6

Table S4 Top 20 most highly abundant transcripts uncovered in the novel transcript discovery analysis. Closest Arabidopsis match determined using BLAST+ protein alignment to TAIR 10 release.

***Brassica napus* transcript ID**	**FPKM**				**Closest Arabidopsis match**	**Putative annotation**
	Westar Mock	Westar 24hpi	ZY821 Mock	ZY821 24hpi		
CUFF.21966.1	2.13	439.75	2.06	3002.7	AT3G22600	Bifunctional inhibitor/lipid-transfer protein/seed storage 2S albumin superfamily protein
CUFF.3931.1	1.08	294.36	1.70	1697.0	AT3G22600	Bifunctional inhibitor/lipid-transfer protein/seed storage 2S albumin superfamily protein
CUFF.4914.2	0.65	171.24	1.00	905.75	AT3G60120	beta glucosidase 27
CUFF.26852.1	9.84	242.26	12.87	898.50	AT3G22890	ATP sulfurylase 1
CUFF.21152.2	0.44	81.63	0.16	367.73	AT2G39200	Seven transmembrane MLO family protein
CUFF.37984.1	359.03	73.62	202.18	231.46	AT2G41430	dehydration-induced protein (ERD15)
CUFF.16712.3	348.21	65.92	270.58	22.97	AT2G39730	rubisco activase
CUFF.22197.12	64.66	158.84	66.36	323.13	AT4G05320	polyubiquitin 10
CUFF.8326.4	95.38	228.50	58.22	181.04	AT4G05320	polyubiquitin 10
CUFF.24223.1	10.36	74.74	9.63	223.57	AT3G62830	NAD(P)-binding Rossmann-fold superfamily protein
CUFF.26562.1	1.21	44.40	0.83	220.17	AT1G30700	FAD-binding Berberine family protein
CUFF.28512.1	8.81	68.41	9.98	208.08	AT1G59870	ABC-2 and Plant PDR ABC-type transporter family protein
CUFF.20400.1	0.35	0.03	0.85	193.79	AT4G01370	MAP kinase 4
CUFF.26855.1	3.31	43.77	3.17	184.54	AT3G22890	ATP sulfurylase 1
CUFF.28029.1	168.87	34.16	51.73	5.91	AT5G09810	actin 7
CUFF.23944.1	83.84	14.48	160.19	5.91	AT2G13770	CONTAINS InterPro DOMAIN/s: Putative harbinger transposase-derived nuclease (InterPro:IPR006912)
CUFF.29327.1	0.28	22.44	0.58	159.48	ATMG00710	Polynucleotidyl transferase, ribonuclease H-like superfamily protein
CUFF.37867.1	1.67	43.79	0.84	148.10	AT3G52400	syntaxin of plants 122
CUFF.4883.1	5.80	50.06	5.11	147.95	AT3G62830	NAD(P)-binding Rossmann-fold superfamily protein
CUFF.35598.2	61.91	63.54	68.60	142.21	AT5G03240	Polyubiquitin 3

Table S6 Transcript levels in FPKM of selected genes. Arabidopsis Gene Identifiers were matched to Brassica napus genes by using BLAST+ protein alignment to the TAIR 10 release.

***Brassica napus* ID**	**FPKM**				**Arabidopsis Gene Identifier**	**Gene name**
	Westar		ZY821			
	Control	24hpi	Control	24hpi		
BnaCnng08300D	7.7	95.0	3.5	15.6	AT1G74710	*ISOCHORISMATE SYNTHETASE 1*
BnaC06g22820D	18.4	61.3	12.6	7.0		
BnaA07g22090D	14.0	23.6	11.8	3.4		
BnaC06g35540D	3.8	20.4	1.9	3.1		
BnaCnng23220D	3.6	0.9	2.8	0.5		
BnaC04g00590D	13.4	6.8	18.3	9.3	AT2G46830	*CIRCADIAN CLOCK ASSOCIATED 1*
BnaA05g01050D	58.1	25.6	57.2	29.9		
BnaA10g00780D	9.5	7.8	35.4	8.8	AT1G01060	*LATE ELONGATED HYPOCOTYL*
BnaC05g00840D	7.8	8.7	30.8	12.4		
BnaAnng17890D	2.8	6.7	15.1	9.3		
BnaC03g00040D	0.9	0.0	12.0	2.8		
BnaA07g15790D	21.6	23.2	12.7	22.0	AT3G52850	*VACUOLAR SORTING RECEPTOR 1*
BnaC06g18530D	58.9	87.0	30.7	284.4	AT3G62420	*BASIC LEUCINE ZIPPER 53*
BnaC08g32220D	42.4	51.1	47.8	218.8		
BnaA09g39870D	60.6	62.8	56.2	135.4		
BnaA07g19330D	5.7	39.5	1.7	5.1		
BnaA07g06750D	0.7	13.0	0.1	236.6	AT3G23230	*ETHYLENE RESPONSE FACROR 98*
BnaA07g33060D	1.2	84.2	2.2	908.7	AT1G76790	*INDOLE GLUCOSINOLATE METHYLTRANSFERASE 5*
BnaC06g37610D	1.6	180.7	1.8	769.5		
BnaC06g21620D	0.9	145.0	1.1	536.9		
BnaC08g44670D	0.5	73.6	2.6	470.3	AT1G06160	*OCTADECANOID-RESPONSIVE ARABIDOPSIS AP2/ERF 59*
BnaA09g50010D	0.6	78.7	2.3	378.6		
BnaC05g04210D	0.0	4.2	0.1	37.0		
BnaA10g04090D	0.0	3.5	0.1	22.2		

Table S9 Primer sequences used for quantitative reverse transcription PCR and genotyping of Arabidopsis mutants.

Species	Gene	Gene Identifier	Direction	Sequence (5' to 3')
Brassica napus	ATGP4	BnaC08g11930D	Forward	CGTCTTCCTCTTCCCTCACC
			Reverse	ACAGTTGGAATAGAATAGTAGGCTC
	LTPG5	CUFF.21966.1	Forward	CCCGAGATATGAACTGAAAGACCT
			Reverse	TGTGCACCTATAAAATTCATACGGC
	MPK4	CUFF.20400.1	Forward	AGATGCTAACCAAGAACACAGA
			Reverse	ACCAGAAAGCTAAACCAAAATGCA
	ACT2	BnaA03g34950D	Forward	TCGAATCTTTCTCGTCCAAGCT
			Reverse	CCAGCGAAACCAGCCTTGA
	TUB4	BnaAnng09960D	Forward	GTTTCGCTCCGTTGACATCG
			Reverse	CGCACATCATGTTCTTCGCA
	APX1	BnaA06g04380D	Forward	CTGCTGACGAGGAAGCATTT
			Reverse	CACACAAGGAACGACACAGC
	RSZ32	BnaA03g00590D	Forward	AGAACAGTCCCAAGAAGCTCAA
			Reverse	ACTAGGACGAGGAGGTGACC
	DORN1	BnaC03g11240D	Forward	TGTGCAAGTACCAGAGAGCAG
			Reverse	GCATTTGTTTGACGAGGCGA
	PDF1.2	BnaA07g32130D	Forward	GCTGCTTTTGAAGCACCAAC
			Reverse	GTTGCAAGATCCATGTCGTG
	PR1	BnaC03g45470D	Forward	TCTCGTTGACCCAAAGGTTC
			Reverse	CAGCCTTCGCTCAAAGCTAC
	SOBIR1	BnaCnng39490D	Forward	CTTTCGTCTCCTCCGTTGAG
			Reverse	TCTCTCGCAGGAAACTCCTC
	MPK9	BnaC02g22230D	Forward	AACGGAAAGTGACCGACAAG
			Reverse	TGAGTCCGGAGAGGTTTGAC
Arabidopsis thaliana	EEF	AT1G30230	Forward	CTGGAGGTTTTGAGGCTGGTAT
			Reverse	CCAAGGGTGAAAGCAAGAAGA
	PR1	AT2G14610	Forward	CTCGGAGCTACGCAGAACAA
			Reverse	CGCTACCCCAGGCTAAGTTT
	PDF1.2	AT5G44420	Forward	CATCATGGCTAAGTTTGCTTCCA
			Reverse	ATTGCCGGTGCGTCGAAA
	VTC 2	AT4G26850	Forward	TCCGACCGTTGTTTCGAACT
			Reverse	CTAGCCCCGTTAAGGCAACA
	DORN1	AT5G60300	Forward	CTCACCCGCTGTCAAAATGG
			Reverse	AGGCTGGACTCTCTGACTGC
