# Supplementary material for: RNA sequencing of Brassica napus reveals cellular redox control of Sclerotinia infection
Source: J Exp Bot. 2017 Sep 27;68(18):5079–91. doi: 10.1093/jxb/erx338 (PMC5853404; doi:10.1093/jxb/erx338)
Supplement: Supplementary-Figures-S1-S4 [file erx338_suppl_supplementary-figures-s1-s4.pdf]

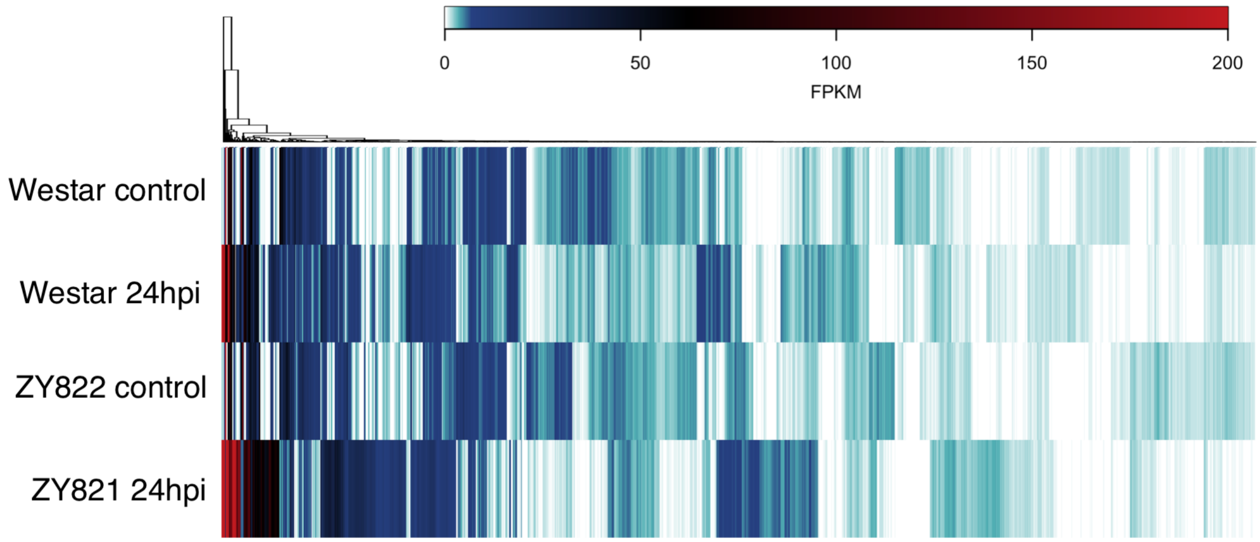

Figure S1 Clustered heatmap of all 1233 newly identified genes based on FPKM levels

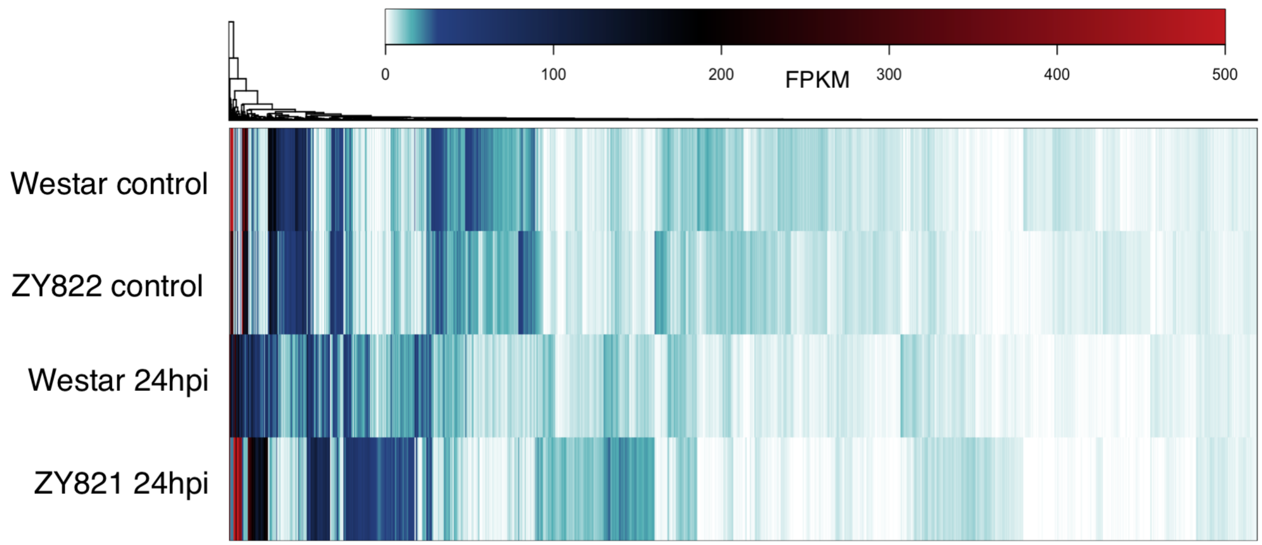

Figure S2 Heatmap of transcript levels in FPKM of all homologues of genes identified as circadian regulated in Arabidopsis (Covington et al., 2008)

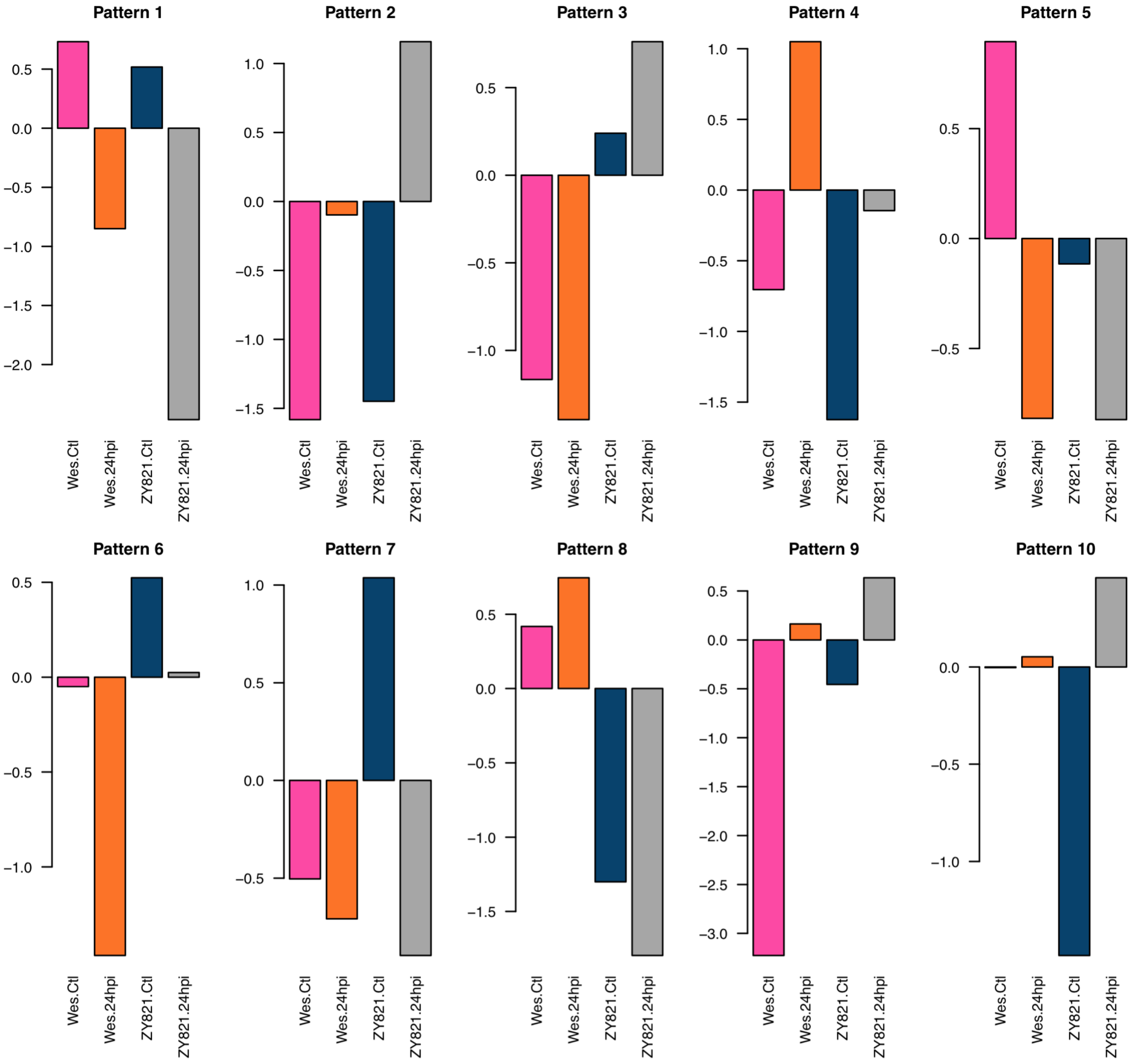

Figure S3 Dominant patterns of gene activity discovered using fuzzy k-means clustering analysis. Bar plots represent relative accumulation level of transcripts belonging to each pattern.

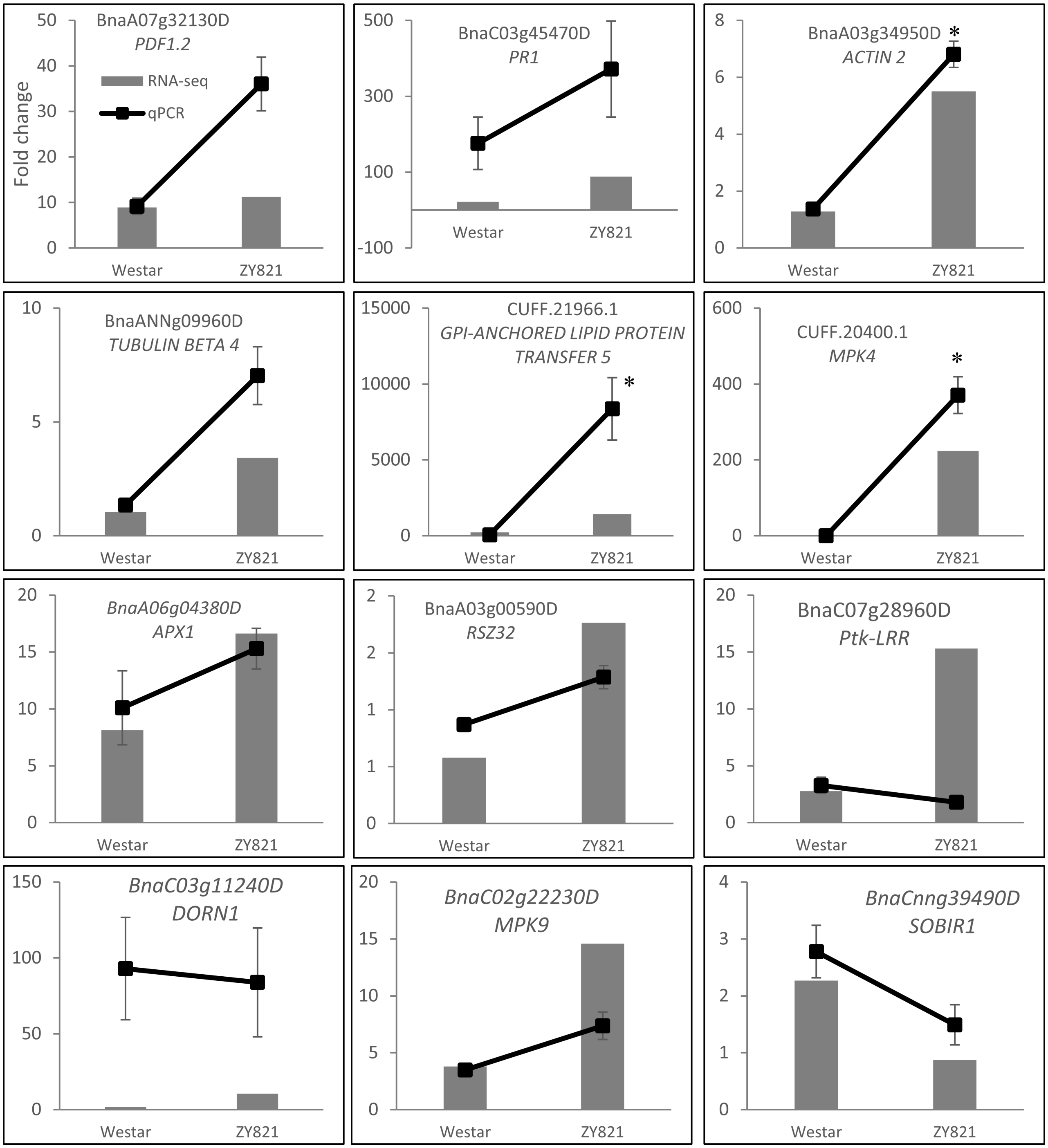

Figure S4 Quantitative reverse transcription PCR of select genes. Relative fold changes from RNA-sequencing data are displayed as bars with qPCR levels as dots.
